# CD34^+^DNAM-1^bright^CXCR4^+^ haemopoietic precursors circulate after chemotherapy, seed lung tissue and generate functional innate-like T cells and NK cells

**DOI:** 10.3389/fimmu.2024.1332781

**Published:** 2024-02-08

**Authors:** Carola Perrone, Federica Bozzano, Maria Giovanna Dal Bello, Genny Del Zotto, Francesca Antonini, Enrico Munari, Enrico Maggi, Francesca Moretta, Alireza Hajabbas Farshchi, Gianluca Pariscenti, Marco Tagliamento, Carlo Genova, Lorenzo Moretta, Andrea De Maria

**Affiliations:** ^1^Experimental Immunology Unit, IRCCS Ospedale Policlinico San Martino, Genova, Italy; ^2^Laboratorio Diagnostico di Autoimmunologia, IRCCS Ospedale Policlinico San Martino, Genova, Italy; ^3^Lung Cancer Unit, IRCCS Ospedale Policlinico San Martino, Genova, Italy; ^4^Integrated Department of Services and Laboratories, IRCCS Istituto Giannina Gaslini, Genova, Italy; ^5^Pathology Unit, Department of Molecular and Translational Medicine, University of Brescia, Brescia, Italy; ^6^Tumor Immunology Unit, Bambino Gesù Children’s Hospital, IRCCS, Rome, Italy; ^7^Department of Laboratory Medicine, Istituto di Ricovero e Cura a Carattere Scientifico Sacro Cuore Don Calabria Hospital, Negrar, Verona, Italy; ^8^Department of Experimental Medicine, University of Genova, Genova, Italy; ^9^Thoracic Surgery Unit, IRCCS Ospedale Policlinico San Martino, Genova, Italy; ^10^Department of Internal Medicine and Medical Specialties (DiMI), University of Genova, Genova, Italy; ^11^Department of Health Sciences, University of Genova, Genova, Italy; ^12^Infections of Immunocompromised Hosts Unit, Division of Infectious Diseases, IRCCS Ospedale Policlinico San Martino, Genova, Italy

**Keywords:** CD34^+^DNAM-1^bright^CXCR4^+^, CD34, NK cell, lung tumor, NSCLC, chemotherapy, immunotherapy, innate-like T-cells

## Abstract

**Background:**

There is little information on the trajectory and developmental fate of Lin^-^CD34^+^DNAM-1^bright^ CXCR4^+^ progenitors exiting bone marrow during systemic inflammation.

**Objective:**

To study Lin^-^CD34^+^DNAM-1^bright^ CXCR4^+^ cell circulation in cancer patients, to characterize their entry into involved lung tissue and to characterize their progenies.

**Methods:**

Flow cytometric analysis of PBMC from 18 patients with lung cancer on samples collected immediately before the first and the second treatment was performed to study Lin^-^CD34^+^DNAM-1^bright^ CXCR4^+^ precursors. Precursors were purified (>99%) and cultured *in vitro* from all patients. Paired PBMC and tissue samples from patients undergoing tumor resection were analyzed by flow cytometry to assess tissue entry and compare phenotype and developmental potential of Lin^-^CD34^+^DNAM-1^bright^ CXCR4^+^ cells in both compartments.

**Results:**

Significant circulation of Lin^-^CD34^+^DNAM-1^bright^ CXCR4^+^ precursors was observed 20d after the first treatment. Precursors express CXC3CR1, CXCR3, CXCR1 consistent with travel towards inflamed tissues. Flowcytometric analysis of lung tissue samples showed precursor presence in all patients in tumor and neighboring uninvolved areas. Successful purification and *in vitro* culture from both blood and lung tissue generates a minor proportion of maturing NK cells (<10%) and a predominant proportion (>85%) of α/β T-progenies with innate-like phenotype expressing NKG2D,NKp30,DNAM-1. Innate-like maturing T-cells *in vitro* are cytotoxic, can be triggered via NKR/TCR co-stimulation and display broad spectrum Th1,Th2 and Th1/Th17 cytokine production.

**Conclusion:**

In advanced stage lung cancer CD34^+^DNAM-1^bright^CXCR4^+^ inflammatory precursors increase upon treatment, enter involved tissues, generate functional progenies and may thus represent an additional player contributing to immune balance in the highly SDF-1/CXCR4-biased pro-metastatic tumor microenvironment.

## Introduction

During the course of inflammatory conditions including HIV, HCV, MTB and COPD, an unconventional CD34^+^DNAM-1^bright^CXCR4^+^ hemopoietic stem cell (HSC) exits the bone marrow (BM) and circulates in peripheral blood (PB) (1). These inflammatory HSCs are common lymphoid precursors (CLP) that express increased proportions of the chemokine receptors CXCR1 and CX3CR1 when compared to PB or cord blood CD34^+^CXCR4^-^DNAM-1^-^ HSCs, and lower proportions of CCR7 and CD62L (1). Accordingly, it has been proposed that they may seed inflamed peripheral tissues where fractalkine/CX3CL1 Eotaxin-3/CCL26 and IL-8/CXCL8 are abundant (2)while still maintaining the ability to seed secondary lymphoid tissues through CCR7 and CD62L signaling.

Under steady-state conditions, in healthy individuals, these HSCs are maintained in the bone marrow (BM) by the homeostatic expression of stromal cell-derived factor 1 (SDF-1; CXCL12) by endosteal and endothelial cells lining bone marrow niches through the interaction of SDF-1 with CXCR4 receptors on HSCs (1, 3–7). Inflammation, however, regulates CXCL12 expression, which acts as BM retention signal for CXCR4^+^ cells in the BM (8), consequently contributing to the release of immature CXCR4^+^ HSCs from the BM (9).

Different from conventional CD34^+^CXCR4^-^ precursors, inflammatory CD34^+^DNAM-1^bright^CXCR4^+^ cells very rapidly generate *in vitro* NK cell and T cell progenies with predominant (2:1) frequency of maturing NK cells (1). In the presence of an HLA-E rich milieu these precursors generate *in vitro* highly functional memory-like NKG2C^+^ NK cell producing IFN-γ and controlling CMV replication (1, 10).

The level of lymphoid infiltration in cancer tissues represents an attempt of the immune response to control the growth of transformed cells in the tumor microenvironment and contributes to the control of tumor spreading (11, 12). Lymphocyte infiltration density correlates inversely to the risk of recurrence in colon cancer (13) and to the response rate to chemotherapy (14). Indeed, tumors with less lymphoid infiltration and with reduced transcript activation with an overall “frozen” transcriptional landscape have worst chances to be controlled by the immune system and by treatments involving checkpoint inhibition (15). In these instances, peripheral blood lymphocyte subsets may also be associated with disease response to immunotherapy (16).

The tumor itself and altered tumor-associated cells may contribute to the production of countermeasures that inactivate infiltrating lymphocyte functions, reduce or dampen inflammation, and divert the tumor infiltrate favoring immune tolerance.

Stem cell circulation in blood after exiting the BM with a trajectory towards peripheral tissues has been well documented in animal models for endothelial- (17), skeletal muscle- (18) and skeleton- (19) tissue-specific stem cells and also for hemopoietic stem cells (20). In humans, tissue seeding of lymphocyte eosinophil progenitors has relevant implications in asthmatic patients during allergen challenge, where they increase in BM, in peripheral blood and in bronchial mucosa (21–25).

There is little information on CD34^+^DNAM-1^bright^CXCR4^+^ Inflammatory precursor entry in inflamed tissues and particularly into cancer tissue, while trafficking and developmental trajectory towards secondary lymphoid organs or thymus of conventional CD34^+^DNAM-1^-^CXCR4^+^ cells is well characterized. In order to address this question, we here studied if and when CD34^+^DNAM-1^bright^CXCR4^+^ cells may be recovered from peripheral blood mononuclear cells (PBMC) of patients with lung tumor and whether they generate progenies with comparable characteristics.

The present work provides insight in timing of inflammatory HSC release from BM in patients with NSCLC, their entry into involved inflamed tissues, and in the characteristics of their progenies.

## Materials and methods

### Patients

For initial analysis of inflammatory precursors, peripheral blood mononuclear cells (PBMC) from 18 cancer patients with progressive disease, encompassing lymphoma, non-small cell lung cancer (NSCLC), and Kaposi’s sarcoma (KS) were collected and compared with healthy donors (n= 18). Additionally, we included samples from HIV patients (n= 15) and COVID patients (n= 28) previously reported in the literature.

Subsequently, we investigated patients with advanced lung cancer scheduled to undergo concurrent chemo-immunotherapy (CT/IT) at baseline, just prior to the initial CT/IT administration (Time 0, T0), and at day 21, immediately before the second CT/IT cycle (Time 1, T1).

For the evaluation of tissue penetration, we analyzed paired blood/tissue samples from five NSCLC patients undergoing surgery according to best practice guidelines. Cancer tissue (C-T) samples and distant uninvolved tissue samples here defined “uninvolved tissue” (U-T) were collected.

Patient characteristics and treatments are indicated in **Table 1**. Sampling was approved by the local ethics committee (registry number: P.R. 191REG2015) and all samples provided informed consent to the observational study.

**Table 1:** Patient characteristics.

ID	GENDER	AGE	STAGE	ISTOTYPE	TREATMENT	DRUGS
1	F	60	IV	Adenocarcinoma	CT+IT	Cisplatin, pemetrexed, pembrolizumab
2	M	71	IV	Adenocarcinoma	CT	Carboplatin, pembrolizumab
3	F	71	IV	Adenocarcinoma	IT	Pembrolizumab
4	M	72	IV	Small cell lung cancer	CT+IT	Carboplatin, etoposide, atezolizumab
5	M	82	IV	Neuroendocrine tumor	IT	Atezolizumab
6	F	72	III	Squamous cell carcinoma	IT	Durvalumab
7	M	66	IV	Adenocarcinoma	IT	Atezolizumab
8	M	79	IV	Adenocarcinoma	IT	Pembrolizumab
9	M	73	IV	Adenocarcinoma	TT	Osimertinib
10	M	79	IV	Adenocarcinoma	CT+IT	Carboplatin, pemetrexed, pembrolizumab
11	F	69	IV	Small cell lung cancer	CT+IT	Carboplatin, etoposide, atezolizumab
12	M	68	IV	Adenocarcinoma	IT	Pembrolizumab
13	M	61	IV	Adenocarcinoma	CT+IT	Carboplatin, pemetrexed, pembrolizumab
14	M	79	IV	Poorly differentiated carcinoma	IT	Pembrolizumab
15	F	57	IV	Squamous cell carcinoma	CT+IT	Carboplatin, paclitaxel, pembrolizumab
16	M	63	IV	Adenocarcinoma	CT+IT	Cisplatin, pemetrexed, pembrolizumab
17	M	59	IV	Squamous cell carcinoma	CT+IT	Carboplatin, paclitaxel, pembrolizumab
18	M	72	IV	Adenocarcinoma	IT	Carboplatin, pemetrexed, pembrolizumab

CT, chemotherapy; IT, immunotherapy; TT, targeted therapy.

### Antibodies

Commercial mouse anti-human mAbs are listed in the **Supplementary Information**. KIR2DL2/L3/S2 (CD158b1/b2,j), anti-KIR3DL1/S1 (CD158e1/e2), anti-KIR2DL1/S1 (CD158a/h), anti-NKG2A (Z270, IgG1; Z199, IgG2a), anti-CD85j (F278, IgG1) were kindly provided by Dr. D. Pende. All were produced in the laboratory (A.Moretta, Genova).

### Cell isolation and culture

PBMC were obtained from peripheral blood by density gradient centrifugation. Samples were cryopreserved until processed. Where applicable, paired samples from the same patient were processed at the same time. Tissue samples were obtained immediately after surgical resection for diagnostic purposes and were selected from Pathology Department of San Martino Hospital, Genoa and Pathology Unit of Sacro Cuore Hospital, Negrar, Verona. Neoplastic and non-neoplastic tissues were mechanically dissociated and cell suspensions obtained were filtered through a 40μm cell strainer (Jet Biofil, Guangzhou, China). Cell suspensions were separated by density gradient centrifugation to obtain PBMCs (Ficoll-Hypaque). Cells were either directly analyzed by flow cytometry or cryopreserved at -86°C for further analyses.

Highly purified Lin^-^CD34^+^DNAM-1^bright^ and CD3^-^CD4^-^CD19^-^CD56^-^CD16^+^ cell populations (>99% purity) were obtained using FACSAria III (BD Biosciences) cell sorter. Purified cells were cultured in limiting dilution conditions in Myelocult medium (StemCell Technologies, Vancouver, British Columbia, Canada) supplemented with 10% human AB serum (ICN Pharmaceuticals Italy, Milano, Italy), 5% FCS and purified recombinant human rhIL-15, rhIL-7, SCF, FLT3-L (PeproThec, London, UK) at the final concentration of 20 ng ml^-1^ with irradiated Feeder cells, for 30 days.

### Immunofluorescence analysis

Cells were analyzed by multi-parameter flow cytofluorometry. Direct staining was performed incubating cells with fluorochrome-conjugated monoclonal antibodies (mAbs) for 15 minutes at 4°C. Cells were then washed and the flow cytometric analysis was performed (FACSFortessa, BD, Mountain View, CA, USA). Mean fluorescence intensity ratios are calculated as follows: MFI sample/MFI negative control and mean fluorescence intensity absolute are calculated as follows: MFI sample-MFI negative control. Data were analyzed using FlowJo (Tree Star, Inc, BD, Ashland, Ore) and FCS Express 7 (*De Novo* Software, Pasadena, Calif).

### Cytokine production assay

CD56-CD3+ progenies derived *in vitro* from purified precursors were plated in 96-well plates (20000 cell/well) in culture medium (RPMI 1620 (BioWhittaker/Lonza) supplemented with 10% FCS, L-glutamine (2 mM), and 1% antibiotic mixture (penicillin–streptomycin 5 mg/mL) and incubated at 37°C overnight.

Maximal stimulus was represented by Phorbol 12-myristate 13-acetate (PMA) (25 ng/mL; Sigma-Aldrich, St Louis, Mo) + ionomycin (1 μg/mL; Sigma-Aldrich). After plate centrifugation, the supernatants were collected and stored at -20°C to measure cytokine release subsequently. Cytokines were measured using a customized MILLIPLEX MAP Human Th17 Magnetic Bead Panel assay (Millipore), using a MAGPIX^®^ with xPONENT^®^ software, according to manufacturer’s instructions. In particular, we analyzed the following cytokines: TNF-α, IFN-γ, IL-2, IL-4, IL-5, IL-9, IL-13, IL-17A, IL-17F, IL-10.

### Cytotoxicity assay

The cytotoxic activity of *in vitro* growing progenies was determined using a PKH-26 and TO-PRO-3 (Sigma-Aldrich and Invitrogen, respectively) assay as previously described (26). FcγR+ P815 mouse mastocytoma and A549 human lung carcinoma cell lines were used as target cells and were labeled with PKH-26 (26). A549 PKH-26^+^ cells were incubated with cell progenies at a 1:1 effector:target (E:T) ratio. Cultures were incubated for 6 h at 37°C in 5% CO2 in complete medium and then placed on ice until flow cytometric analysis.

Cytotoxic activity against P815 cells was tested in a reverse ADCC (Ab-dependent’ cell-mediated cytotoxicity) at a 1:1 effector:target (E:T) ratio in complete medium in the absence or presence of mAbs (0.1 μg/mL). P815-PKH-26^+^ cells were incubated with effector cells for 6 h at 37°C in 5% CO2 in complete medium and then placed on ice until flow cytometric analysis. Spontaneous and maximal target cell deaths were determined by PKH-26 labeling of cells cultured alone and permeabilized with BD Cytofix/Cytoperm reagent (BD Pharmingen), respectively. To identify dead cells, 5 μl of a 10 μM stock solution of TO-PRO-3 was added to each tube immediately before analysis. Cells were analyzed by FACSCFortessa (BD), and 10.000 events were collected. Specific lysis was calculated by use of the following formula for dye-labeled cells: (sample − spontaneous)/(total − spontaneous) × 100.

### Statistical analysis

Statistical analysis was performed using the Mann-Whitney U test for unpaired datasets for comparisons. Analysis was performed using JMP 10.0 (SAS) if not otherwise stated. Significant differences (two-tailed) are reported in the text and figures, while non-significant differences are reported in the figure legends only.

## Results

### Normal baseline and increased CD34^+^DNAM-1^bright^CXCR4^+^ precursor circulation after chemotherapy/immunotherapy in lung cancer patients

We first verified whether CD34^+^DNAM-1^bright^CXCR4^+^ “inflammatory” CLP that are detected in PBMC of patients with acute or chronic infections (1, 27, 28) could also be detected in cancer patients. We investigated PBMC of sequential patients on treatment for non-Hodgkin lymphoma, NSCLC, Kaposi’s sarcoma (KS).

**Figure 1:**
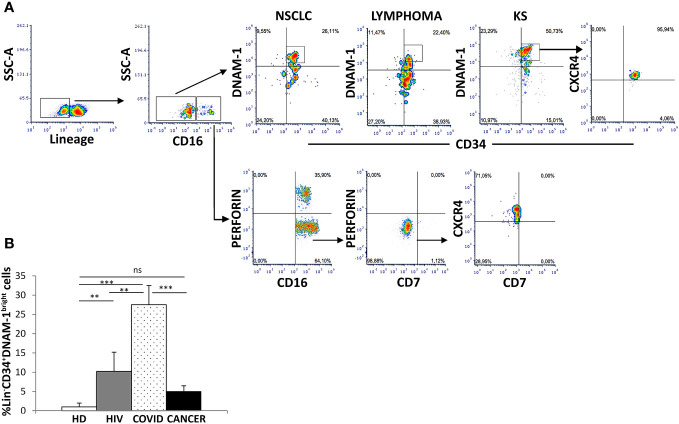
Identification of inflammatory precursors in PB of cancer patients by flow cytometry. **(A)** inflammatory precursors (Lin^−^CD34^+^DNAM-1^bright^CXCR4^+^ and Lin^-^CD56^-^CD16^+^CD7^-^CXCR4^+^ are recovered from PBMC of patients with different tumors (representative of 3 patients each cancer type). Flow cytometric gating strategy to identify and characterize CXCR4+ CLPs in cancer PBMC. Among Lineage^-^ (CD3^-^CD14^-^CD19^-^CD20^-^CD56^-)^ (Lin^-^) gated cells Lin^−^CD34^+^DNAM-1^bright^ and Lin^-^CD56^-^CD16^+^CD7^-^ cells are shown. **(B)** Frequency of Lin^−^CD34^+^DNAM-1^bright^ precursors in patients with ART-treated HIV infection (HIV) (#18, white box), symptomatic COVID (COVID) (#28, white dotted box) and untreated Lung Cancer patients (#18, black box) at diagnosis before CT compared to healthy donors (HD) (#18, white box). (COVID vs. HD, ***p<0.0001; COVID vs. HIV, **p=0.0036; COVID vs. CANCER, ***p=0.0001; HIV vs. HD, **p=0.0020; CANCER vs. HD, p=0,1 ns; Mann-Whitney U-test). Histograms show mean ± SD. SSC-A, side scatter area.

Using a reverse flow cytometric gating strategy with Lin^-^ selection stain (which includes anti-CD3, -CD19, -CD20, -CD14, -CD16, -CD56 mAbs) Lin^-^CD34^+^DNAM-1^bright^ precursor cells could be detected in all the patients with non-Hodgkin Lymphoma, NSCLC and Kaposi Sarcoma (**Figure 1A**). In addition, within the same gating strategy also Lin^-^CD56^-^CD16^+^CD7^-^ cells could be detected corresponding to a different previously described CLP (10) (**Figure 1A**). Both precursor cell populations circulating in PBMC of cancer patients expressed CXCR4, in line with their recent exit from BM upon inflammation (1)(**Figure 1A**).

In order to study the dynamic evolution of inflammatory precursor circulation in cancer patients, we next studied PBMC of a new cohort of 18 patients with newly diagnosed advanced lung cancer by flowcytometry with sampling at diagnosis before any systemic treatment and after 21 days just before the second treatment. Demographic and clinical characteristics are reported in **Table 1**.

When compared to HDs, no difference in the circulation of Lin^-^CD34^+^DNAM-1^bright^CXCR4^+^ cells was detected in cancer patients at baseline (**Figure 1B**). The frequency of Lin^-^CD34^+^DNAM-1^bright^ cells was also compared to COVID-19 or to chronic HIV-1 patients, where relevant increases in circulating inflammatory CLP occur. Increased precursor frequencies were confirmed during SARS-CoV-2 and HIV-1 patients compared to lung cancer patients at baseline (27,5 ± 24,07% vs. 10,2 ± 1,4 vs. 5,07 ± 1,4; SARS-CoV-2, HIV-1 and lung cancer respectively; p=0.0036, p=0.0001 U-test, for comparison with cancer patients, respectively; **Figure 1B**).

**Figure 2:**
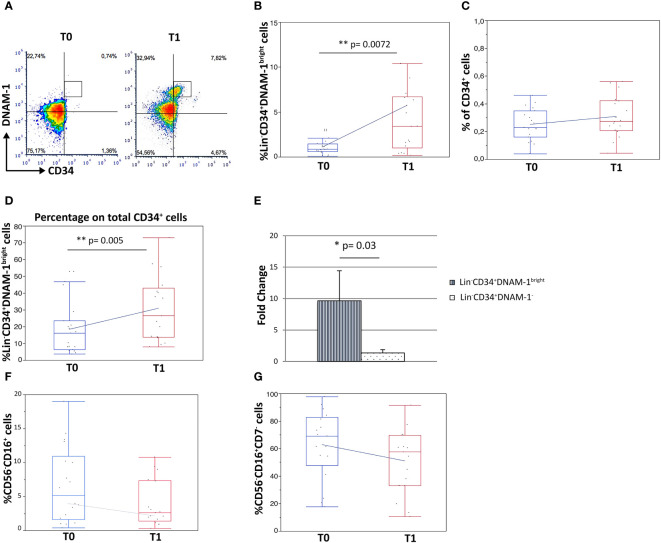
Increased frequency of Lin^-^CD34^+^DNAM-1^bright^ and Lin^-^CD56^-^CD16^+^CD7^-^ precursor cells in peripheral blood of lung cancer patients following treatment. Experiments represent 2 time points, before therapy (T0) and 21 days after the first cycle of therapy (T1), for each of 18 cancer patients. **(A)** Dot plots show flow cytometric analysis of Lin^−^CD34^+^DNAM-1^bright^ cells in patient PBMCs at T0 and T1. **(B)** Box-plot representation of the proportion of Lin^−^CD34^+^DNAM-1^bright^ in peripheral blood of cancer patients. T1 vs. T0 (**p=0.007) (#18 patients). **(C)** Box-plot representation of CD34^+^ cell frequency in peripheral blood of cancer patients before and after therapy (T0: 0,23 ± 0,12%; T1: 0,31 ± 0,15%) (#18 patients) (p=n.s.). **(D)** Box-plot representation of the increase of Lin^−^CD34^+^DNAM-1^bright^ cells over total CD34^+^ cells at T1 (15,3 ± 10,8% vs. 29,3 ± 19,9% mean ± sem; (**p=0.005, U-Test) (#18 patients). **(E)** Fold-change increase at T1 over T0 of Lin^−^CD34^+^DNAM-1^bright^ cell frequency in PB is higher compared to fold-change of Lin^-^CD34^+^DNAM-1^-^ cells (*p=0.03, Mann-Whitney U-test) (#18 patients). Histograms indicate mean ± sem. **(F, G)** Box-plot representation of Lin^-^CD56^-^CD16^+^ and Lin^-^CD56^-^CD16^+^CD7^-^ cells frequency. No significant difference in percentage before vs. after therapy were detected (#18 cancer patients). Mann-Whitney U-test; no symbol corresponds to: p=n.s.).

Analysis by flow cytometry of paired PBMC samples collected immediately before therapy (T0) and before the second cycle of therapy (21 days: T1) on the contrary revealed a 5-fold increase in Lin^-^CD34^+^DNAM-1^bright^ cell frequency after CT/IT (**Figures 2A, B**).(1,11 ± 1,10 vs. 5,68 ± 2,07 median ± DS, T0 vs. T1 respectively; p=0.0072; **Figure 2B**).

In order to determine whether the observed increase was part of a generalized mobilization of CD34 cells from the BM, or rather an event restricted to inflammatory precursors, the fractional frequency of conventional and of inflammatory CD34^+^ cells over total CD34^+^ precursors were considered. Interestingly, total circulating CD34^+^ cell frequencies were not increased following CT/IT (**Figure 2C**), while the proportion of CD34^+^DNAM-1^bright^ cells significantly increased after CT/IT (15,31 ± 10,8 vs. 29,36 ± 19,9; T0 vs. T1; p=0.005; **Figure 2D**). These findings indicating a selective BM exit of Lin^-^CD34^+^DNAM-1^bright^ precursors (**Figure 2B**) was also confirmed by a higher fold-change in their frequency after CT/IT compared to classical CD34^+^DNAM-1^-^ (p=0.03; **Figure 2E**).

In addition, the frequency of CD34^-^Lin^-^CD56^-^CD16^+^CD7^-^CXCR4^+^ precursors that are enriched in PBMC during CMV reactivation (10, 29) and of the Lin^-^CD16^+^CD56^-^ subset where they appear mixed with otherwise exhausted CD16^+^CD56^-^ NK cells, was unchanged (**Figures 2F, G**).

Overall, therefore, these data indicate that in patients with lung tumor only CT/IT induces selective increase of specialized CD34^+^DNAM-1^bright^CXCR4^+^ precursors.

### Identification of Lin^-^CD34^+^DNAM-1^bright^CXCR4^+^ cells in lung tissue samples from NSCLC patients

Circulating Lin^-^CD34^+^DNAM-1^bright^ cells have been shown to have a different expression of chemokine receptors when compared to canonical CD34^+^DNAM-1^-^ cells that would support their migration into inflamed tissues (1).

In this regard, although DNAM-1 is known as an activating receptor constitutively expressed by NK cells, T cells, macrophages, and DCs [(30) specifically recognizing two cell ligands (PVR, CD155 and Nectin 2, CD112] (31), it also is crucially involved in transendothelial cell migration (32). Expression of high DNAM-1 densities on inflammatory precursors therefore could contribute to transendothelial cell migration of Lin^-^CD34^+^DNAM-1^bright^CXCR4^+^ cells out of BM niches and also into inflamed tissues possibly leading to is down-expression upon tissue entry.

**Figure 3:**
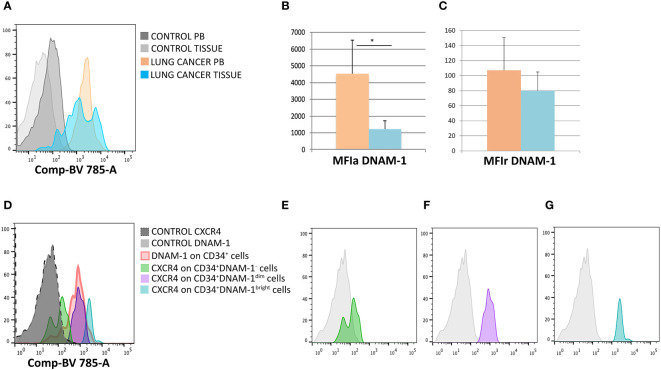
DNAM-1 and CXCR4 expression on peripheral blood and -tissue CD34^+^ cells. **(A)** Expression of DNAM-1 on peripheral blood (PB) CD34^+^cells of lung cancer patients (in orange) and lung cancer-tissue CD34^+^ cells (in light blue) in a representative patient; negative controls (of PB and TISSUE) are shown in gray. Flowcytometric Histogram representation. **(B)** Group comparison of DNAM-1 molecule density on CD34^+^ inflammatory precursors circulating in blood and in tissue shows higher DNAM-1 MFIa expression in peripheral blood (orange column) compared to lung cancer-tissue (light blue column) (*p=0.0314, mean ± sem,Mann-Whitney U-test). **(C)** Group comparison of DNAM-1 molecule density on CD34^+^ inflammatory precursors circulating in blood and in tissue. MFIr (MFI corrected for negative control) expression in peripheral blood (orange column) compared to lung cancer-tissue (light blue column). (no symbol corresponds to: p=n.s., mean ± sem,Mann-Whitney U-test)). **(D)** Molecule density (MFI) of CXCR4 expression on CD34^+^DNAM-1 precursors by flow cytometry according to the expression of DNAM-1 (DNAM-1^-^; DNAM-1^dim;^ DNAM-1^bright^) in CD34^+^ precursors in cancer-tissue samples. (Representative of 5 patients). **(E–G)** CXCR4 molecule density by flow cytometric analysis according to DNAM-1 MFI. Lin^-^CD34^+^DNAM-1^-^, Lin^-^CD34^+^DNAM-1^dim^ and Lin^-^CD34^+^DNAM-1^bright^ cells of cancer-tissue samples, respectively (Representative of 5 patients).

To address these questions we therefore analyzed parallel samples of tumor tissue, uninvolved lung tissue and PBMC from 5 patients undergoing surgery for lung cancer CD34^+^DNAM-1^bright^CXCR4^+^ cells could be identified, in addition to PBMC, in all tissue samples. Analysis of DNAM-1 MFI in CD34^+^DNAM-1^bright^CXCR4^+^ cells (**Figure 3A**) showed a higher mean DNAM-1 density in peripheral blood vs. cancer-tissue precursors (p=0.03; **Figure 3B**). When analyzing MFI with negative control correction (MFIr), the difference in DNAM-1 molecule density was more comparable in blood and in tissue samples. (**Figure 3C**).

Since Lin^-^CD34^+^DNAM-1^bright^CXCR4^+^ cells are also identified by their CXCR4 expression, we additionally studied CXCR4 expression by DNAM-1 MFI, to confirm the presence of these cells in the samples. In lung tissues, but not in PB, DNAM-1 molecule density could identify 3 subsets of cells including Lin^-^CD34^+^DNAM-1^bright^, Lin^-^CD34^+^DNAM-1^dim^cells, Lin^-^CD34^+^DNAM-1^-^ cells (**Figure 3D**). Lin^-^CD34^+^DNAM-1^-^ cells did not express CXCR4 (**Figure 3E**) while Lin^-^CD34^+^DNAM-1^bright^ and Lin^-^CD34^+^DNAM-1^dim^cells both expressed CXCR4, with higher molecule density on the former (**Figures 3F, G**). Whole tissue analysis thus indicates that Lin^-^CD34^+^DNAM-1^bright^CXCR4^+^ cells- but not conventional CD34+ precursors- enter and can be recovered in tumor tissue.

### Peripheral inflammatory precursors in cancer patients are oriented towards inflamed tissues according to chemokine receptor expression

We next analyzed the frequency of Lin^-^CD34^+^DNAM-1^bright^CXCR4^+^cells recovered in blood, tumor and uninvolved tissue samples in order to provide an estimate of their tissue trafficking.

**Figure 4:**
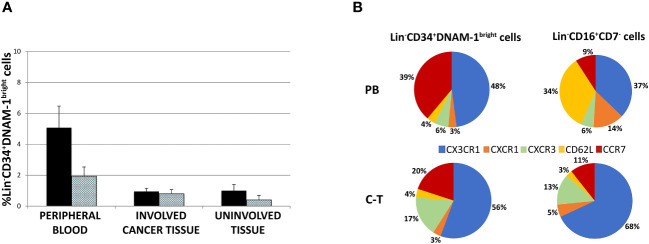
**(A)** Lin^-^CD34^+^DNAM-1^bright^ cell frequency in peripheral blood of cancer patients (#18 samples), lung cancer-tissue (#15 samples) and lung uninvolved tissue (#14 samples). Histograms show mean ± sem. CD34^+^DNAM-1^bright^ cells (Black column); CD34^+^DNAM-1^bright^ cells in lymphocyte gate (shaded column). no symbol corresponds to: P= n.s. U-test for all comparisons) **(B)** Chemokine receptor pie chart analysis of CLPs derived from peripheral blood (PB) and tissue (C-T) of cancer patients. Number express the mean relative frequency of expression.

The frequency of peripheral blood Lin^-^CD34^+^DNAM-1^bright^CXCR4^+^cells was higher compared to tissue Lin^-^CD34^+^DNAM-1^bright^CXCR4^+^ cells, while no difference was observed between involved and uninvolved tissue (**Figure 4A**). Cell migration into tissues follows chemokine gradients and chemokine receptor expression governs lymphocyte trafficking into tissues. Accordingly, different chemokine receptor expression by these cells could associate with differences in observed PB vs. tissue frequencies.

Flow cytometric analysis of chemokine receptor expression by Lin^-^CD34^+^DNAM-1^bright^CXCR4^+^ or Lin-CD56^-^CD16^+^CD7^-^ cells showed that relevant differences are detectable in the peripheral blood in a representative patient with predominant expression of CX3CR1 (89-94%), lower levels of CXCR1 (25-28%), only minor expression of CXCR3 (10-18%) and of CD62L (4-10%), and expression of CCR7 (60-65%) (**Supplementary Figures 1A, B**). This pattern of expression suggests their potential ability to migrate towards inflamed tissues following CX3CR1:fractalkine and CXCR3:IL-8 gradients in addition to their possible transit towards secondary lymphoid tissues via CCR7.

The same analysis was also performed on Lin^-^CD56^-^CD16^+^CD7^-^ precursors. Also, in this case we observed expression of CX3CR1 (93-98%) and CXCR1 (23-27%), little CXCR3 (4-6%), a consistent expression of CD62L (46-52%) and a minor expression of CCR7 (10-16%) (**Supplementary Figures 1A, B**).

Thus, when considering these CLPs in PB, expression of CX3CR1 and CCR7 predominates on Lin^-^CD34^+^DNAM-1^bright^CXCR4^+^ cells with low levels of CXCR1, CXCR3, CD62L, and CCR7. On the other hand, Lin^-^CD56^-^CD16^+^CD7^-^ cells display a different chemokine receptor signature, with predominant expression of CX3CR1 and CD62L and lower expression of CXCR1 and (**Figure 4B** and **Supplementary Figures 1A, B**). Overall, the frequency of CCR7 and CD62L expression on Lin^-^CD34^+^DNAM-1^bright^CXCR4^+^ in PBMC (40-50%) suggests that this proportion could enter secondary lymphoid organs (**Figure 4B**), thus accounting for a reduced fractional recovery in tissues compared to PB.

To verify this possibility, chemokine receptor expression was studied by flow cytometry also on common lymphocyte precursors recovered from tissues. Both Lin^-^CD34^+^DNAM-1^bright^ cells and Lin-CD56^-^CD16^+^CD7^-^ tissue cells were recovered in patient tissues and were enriched for the expression of chemokine receptors driving towards inflamed tissues. Indeed, their combined mean expression of CCR7 or CD62L accounted for only 24% and 14% respectively, with correspondingly increased frequencies of CX3CR1, CXCR3 and CXCR1 expression (**Figure 4B**). Thus inflammatory precursors from PBMC expressing CXCR1, CXCR3 and CX3CR1 selectively enter inflamed tumor tissues.

### Purification, culture and progeny characterization of CLP from tissues and peripheral blood in cancer patients

**Figure 5:**
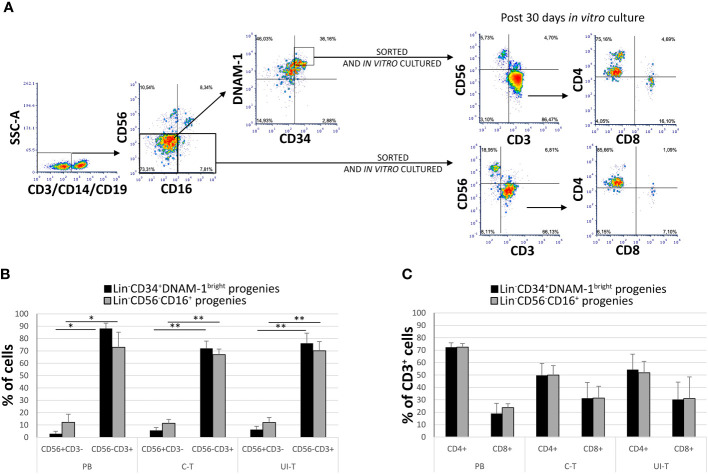
*In vitro* culture of highly purified Lin^-^CD34^+^DNAM-1^bright^ and Lin^-^CD56^-^CD16^+^ cells from peripheral blood (PB), cancer tissue (C-T) and uninvolved tissue (UI-T) of lung cancer patients generates NK and T progenies. **(A)** Flow cytometric purification strategy and culture of Lin^-^CD34^+^DNAM-1^bright^ and Lin^-^CD56-CD16+ cells isolated from PB, C-T and UI-T. **(B)** Characterization of *in vitro* cultures from highly purified Lin^-^CD34^+^DNAM-1^bright^ (black columns) and Lin^-^CD56^-^CD16^+^ (grey columns) cells from PB, C-T and UI-T. Bars express the proportion of cells recovered in culture. Maturing T cell progenies are predominant in *in vitro* cultures derived from both CLPs. Histograms (mean ± sem) show 18 different cultures derived from each of the two CLP populations isolated from 4 different PB, 7 different C-T and 7 different UI-T samples. Mann-Whitney U-test analysis is shown. **(C)** Expression of CD4 and CD8 surface molecules on CD56^-^CD3^+^ T cell progenies derived from highly purified Lin^-^CD34^+^DNAM-1^bright^ (black columns) and Lin^-^CD56^-^CD16^+^ (grey columns) cells from PB, C-T and UI-T. Histograms (mean ± sem) show 18 different cultures derived from each of the two CLP populations isolated from 4 different PB, 7 different C-T and 7 different UI-T patients. (no symbol corresponds to: p=n.s.; Mann-Whitney U-test, all comparisons). SSC-A, side scatter area. “**” indicates a p value < 0.01, “*” indicates a p value < 0.05.

In patients with chronic infections, the precursors identified as Lin^-^CD34^+^DNAM-1^bright^ and Lin^-^CD56^-^CD16^+^CD7^-^ both generate *in vitro* NK cell progenies with a minor (25%) frequency of T cell progenies (10). However, no information has been available on the behavior of these precursors when they migrate into tissues. Therefore, following the isolation of these inflammatory precursors from both peripheral blood (PB) and lung tissue in patients with NSCLC, we conducted a comparative characterization of their progenies. Tissue mononuclear cell preparations were obtained from both uninvolved lung tissue (U-T) and cancer tissue (C-T) samples derived from seven different patients, along with corresponding PB samples. Highly purified cells (**Figure 5A** details the gating strategy) were then cultured *in vitro* under limiting dilution conditions.

Progenies were identified optically for growth after 16-20days from purification and seeding, and subsequently analyzed after 30 ± 1days (mean ± SEM). Quantitatively sufficient growing cultures were obtained from all patients from purified Lin^-^CD34^+^DNAM-1^bright^ including 45 cultures from C-T, 43 cultures from U-T and 59 cultures from PB. Similarly, from highly purified Lin^-^CD56^-^CD16^+^CD7^-^ precursors we obtained 33 cultures from C-T, 45 from U-T and 61 from PB. Flow cytometric analysis showed that precursor cultures were composed of lymphoid cells that were in majority CD56^-^CD3^+^ (85-90%) with lower frequency of CD56^+^CD3^-^ (<10%) and a minor representation of CD56^+^CD3^+^ cells (4%) which were not further studied (**Figure 5B**).

**Figure 6:**
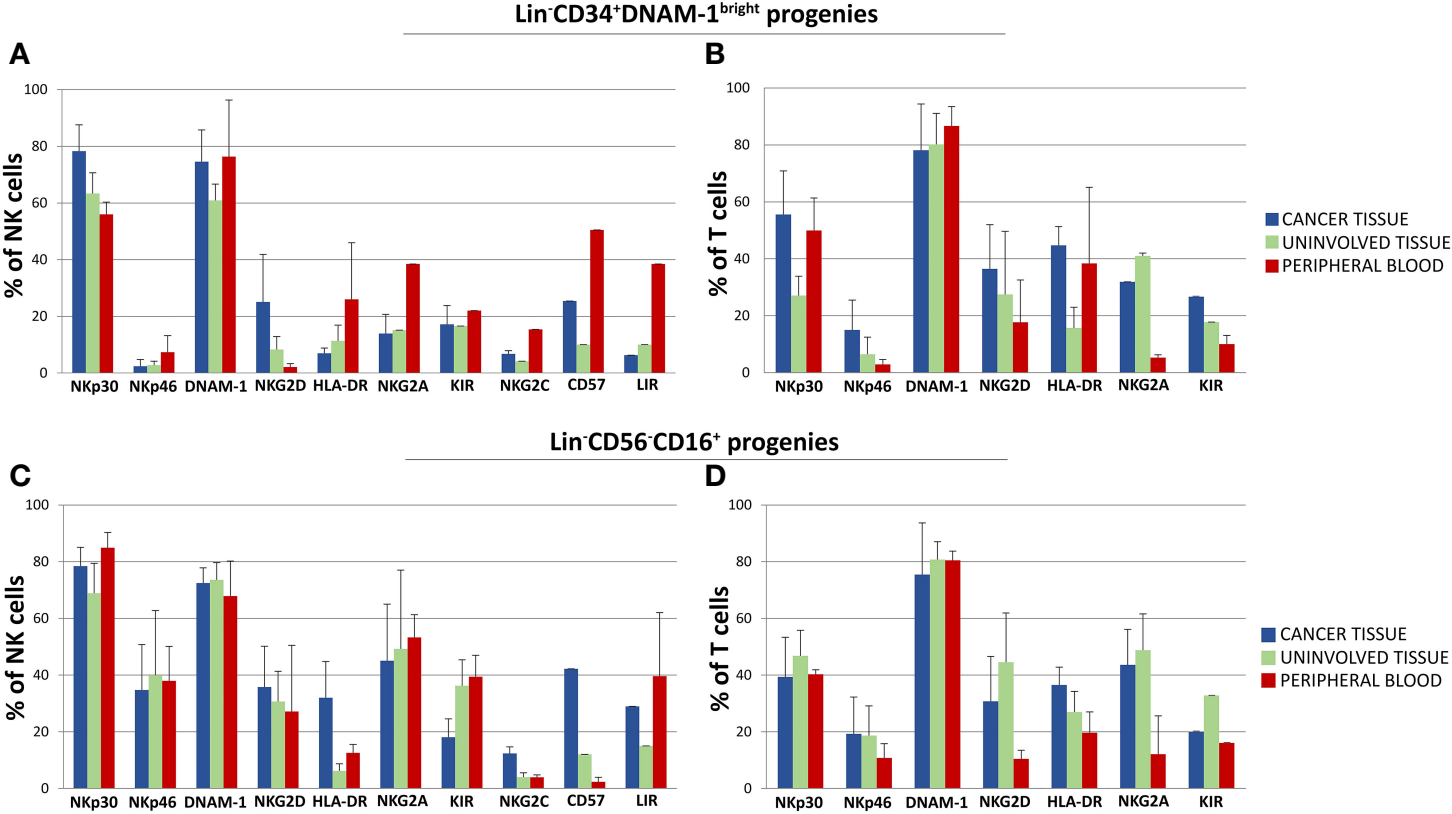
Phenotypic characterization by flow cytometry of *in vitro* developing NK and T cell progenies derived from highly purified Lin^-^CD34^+^DNAM-1^bright^ and Lin^-^CD56^-^CD16^+^ cells from cancer tissue (C-T), uninvolved tissue (UI-T) and peripheral blood (PB) from the same patients with lung cancer. **(A, B)** Mean Frequency of expression of given markers by NK **(A)** and T cell **(B)** progenies from highly purified C-T (blue columns), UI-T (green columns) and PB (red columns) Lin^-^CD34^+^DNAM-1^bright^ cells. Histograms (mean ± sem) -(#5 C-T experiments; #5 UI-T experiments; #4 PB experiments), p=n.s. (Mann-Whitney U-test, for all comparisons). **(C, D)** Mean Frequency of expression of given markers by NK (Panel **C**) and T cell **(D)** progenies from highly purified C-T (blue columns), UI-T (green columns) and PB (red columns) Lin^-^CD56^-^CD16^+^ cells. Histograms indicate mean ± sem. (#5 C-T experiments; #5 UI-T experiments; #4 PB experiments), no symbol corresponds to: p=n.s. (Mann-Whitney U-test, for all comparisons).

In comparing progenies derived from different purified precursors, NK cells constituted a substantial minority. Indeed, <10% of all proliferating cultures from purified CD34^+^DNAM-1^bright^CXCR4^+^ cells and <15% of those from Lin^-^CD56^-^CD16^+^CD7^-^CXCR4^+^ cells were NK cells (**Figure 5B**). T cell progenies overwhelmingly dominated cultures from both precursors in all three conditions (PB, C-T, U-T). Within maturing T cell cultures, the frequency of CD4^+^ T cells cells surpassed that of CD8^+^ T cells (**Figure 5C**). NK and T cell progenies were further investigated using flow cytometry, specifically focusing on the expression of activating and inhibitory receptors NK cell progenies derived *in vitro* from purified CD34^+^DNAM-1^bright^CXCR4^+^ or Lin^-^CD56^-^CD16^+^CD7^-^CXCR4^+^ cells expressed comparable frequencies of NKp30, DNAM-1, KIRs (67,1 + 3,5% vs.76,3 ± 3,35%; 69,8 ± 4.5% vs. 71,8 ± 2,6%; 17,9 ± 1,6% vs. 29.2 ± 3,5% Mean ± SEM respectively) and a decreased frequency of NKG2A expression (19,3 + 1,6% vs 48,7 + 6,6% Mean ± SEM p=0.0053) (**Figures 6A, C**). In addition, consistent frequencies of surface molecules that are expressed by adaptive NK cells were detected in progenies derived from both CLPs isolated from tissues (NKG2C 7,4 ± 1,4% vs. 7,1 ± 1,2%; CD85j 16.2 ± 4.9% vs. 27.6 ± 6.0%; CD57 23.9 ± 6.3% vs. 14.1 ± 4.9%, Mean ± SEM, respectively). Lower frequencies of NKp46 and NKG2D expression was observed on progenies from CD34^+^DNAM-1^bright^CXCR4^+^ cells compared to those derived *in vitro* from Lin^-^CD56^-^CD16^+^CD7^-^CXCR4^+^ cells (3.9 ± 1.8% vs. 37.3 ± 6-9% and 13.0 ± 4.6% vs. 32.1 ± 6.6% Mean ± SEM p=0.0019 and p=0.024, respectively). Due to small cell numbers, no functional test could be performed on NK cell progenies.

The majority of progenies derived from inflammatory precursors both in peripheral blood mononuclear cells (PBMCs) and tissue samples were T cells. These T-cell progenies exhibited high levels of activating NK-cell receptors, including NKp30 (30-50%), NKp46 (10-20%), NKG2D (15-25%), and DNAM-1 (75-85%). Importantly, progenies from both CD34^+^DNAM-1^bright^CXCR4^+^ and Lin^-^CD56^-^CD16^+^CD7^-^ purified cells derived from cancer tissue, uninvolved tissue, and PBMC consistently expressed inhibiting NK cell receptors (KIRs and NKG2A) (**Figures 6B, D**).

**Figure 7:**
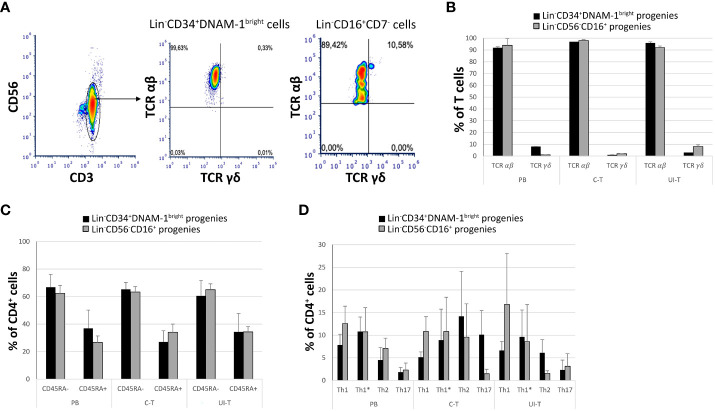
Characterization of *in vitro* derived T progenies from CLP cells derived from tissue samples and peripheral blood of cancer patients. PB (peripheral blood); C-T (cancer tissue); UI-T (uninvolved tissue) by flow cytometry. **(A)** Flow cytometric analysis of TCR molecule expression on PB, C-T and UI-T Lin^-^CD34^+^DNAM-1^bright^ and on Lin^-^CD56^-^CD16^+^ derived T cell progenies grown *in vitro*. CD3^+^CD56^-^ gated cells were studied for the expression of TCR *αβ* and *γδ* after 30 days from purification of precursor cells. Representative of 4 experiments. **(B)** Frequencies of TCR *αβ* or *γδ* expression on T-cell progenies derived from PB, C-T and UI-T Lin^-^CD34^+^DNAM-1^bright^ (black columns) and Lin^-^CD56^-^CD16^+^ (grey columns) cells. Histograms indicate mean ± sem; n= 4 experiments. p=n.s. (Mann-Whitney U-test, for all comparisons). **(C)** Expression of CD45RA surface molecule on CD4^+^ T-cell progenies derived from PB, C-T and UI-T Lin^-^CD34^+^DNAM-1^bright^ (black columns) and Lin^-^CD56^-^CD16^+^ (grey columns) cells. (#4 PB samples; #6 C-T samples; #6 UI-T samples). Histograms indicate the mean ± sem. p=n.s. (Mann-Whitney U-test, for all comparisons). **(D)** Frequency of T helper cell subsets (Th1, Th1*, Th2 and Th17) CD4^+^ progenies *in vitro* derived from PB, C-T and UI-T Lin^-^CD34^+^DNAM-1^bright^ (black columns) and Lin^-^CD56^-^CD16^+^ (grey columns) cells. Histograms indicate mean ± sem. SSC-A, side scatter area. no symbol corresponds to: p=n.s. (Mann-Whitney U-test, for all comparisons).

Additionally, T-cell progenies predominantly expressed the αβ T cell receptor (TCRαβ), with only a minor frequency of the γδ TCR (<2-3%) (**Figures 7A, B**). Analysis of CD45RA expression on CD4^+^ cells revealed a bimodal distribution, with a higher proportion of CD4^+^CD45RA^-^ cells and a consistent but smaller fraction of CD4^+^CD45RA^+^ cells across all samples and patients (**Figure 7C**).

To further characterize the T helper (Th) differentiation potential of CD4^+^CD45RA^-^ progenies, we employed flow cytometry to assess their expression of Th1, Th2, and Th17-associated markers, including CXCR3, CCR4, CCR6, and CCR10 according to a previously described method with a gating strategy depicted in **Figure 7D**. Accordingly, we successfully distinguished various T helper (Th) subsets, including also Th1*. These Th1* cells, as defined by Sallusto represent a unique subset of Th cells that exhibit a dual phenotype, combining characteristics of both Th1 and Th17 cells. Notably, Th1* cells express CXCR3 and produce IFN-γ, two hallmarks of Th1 cells, while also expressing CCR6, a marker typically associated with Th17 cells. However, in contrast to Th17 cells, Th1* cells do not produce IL-17 (33, 34).

CD3^+^CD4^+^CD45RA- maturing progenies *in vitro* derived from C-T Lin^-^CD34^+^DNAM-1^bright^ cells were Th1, Th1*, Th2, Th17 (5,1 ± 1,1; 8,9 ± 6,8; 14,2 ± 9,9; 10,1 ± 5,3 respectively (mean ± sem) and from C-T Lin-CD56-CD16+ cells [10,8 ± 3,2; 10,7 ± 7,6; 9,5 ± 7,3; 1,4 ± 1 respectively (mean ± sem)].

CD3^+^CD4^+^CD45RA^-^ maturing progenies *in vitro* derived from UI-T Lin^-^CD34^+^DNAM-1^bright^ cells were Th1, Th1*, Th2, Th17 [6,6 ± 1,9; 9,6 ± 5,9; 6,1 ± 2,9; 2,3 ± 2,1 respectively (mean ± sem)] and from UI-T Lin^-^CD56^-^CD16^+^CD7^-^ cells were mainly Th1 and Th1* with lower frequency of Th2 and Th17 cells [16,8 ± 11,2; 8,5 ± 7,2; 1,5 ± 0,5; 3,1 ± 2,7 respectively (mean ± sem)] (**Figure 7D**). In general therefore, a consistent pattern of potential Th1 and Th17 cytokine production potential emerged, with however a wide spectrum of helper type progenies.

### Maturing progenies from inflammatory CLP migrated to tumor tissue are functionally comparable to those of CLP purified from PBMC in NSCLC patients

In light of the diverse array of T helper cell (Th) subsets identified among CD4+ T cell progenies based on their chemokine receptor expression, we assessed the actual cytokine secretion capabilities of these progenies. Culture supernatants were evaluated either without additional stimulus beyond culture conditions (“unstimulated”) or after additional activation with PMA+ionomycin (“stimulated”).

**Figure 8:**
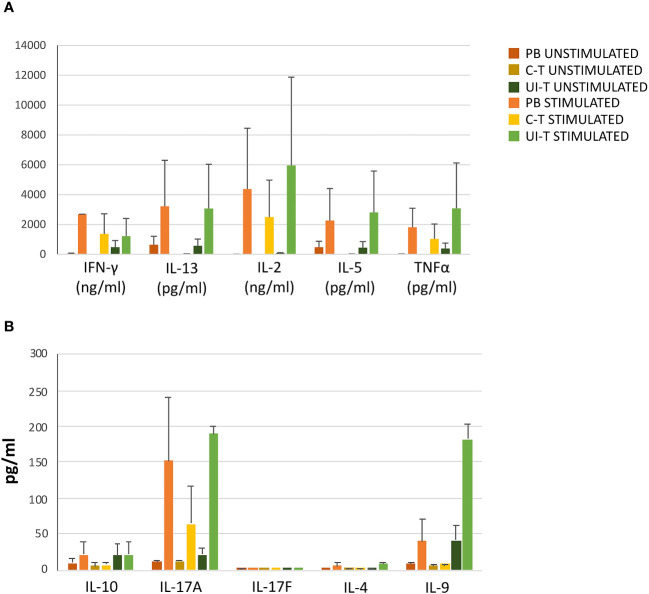
Cytokine production by T-cell progenies derived *in vitro* from CLP cells isolated from peripheral blood (PB), cancer tissue (C-T), UI-T (uninvolved tissue) CLP cells. Histograms in **(A, B)** show cytokine production of *in vitro* derived T progenies from PB, C-T and UI-T CLPs in culture medium without stimulation (dark orange, dark yellow and dark green bars respectively) and with phorbol 12-myristate 13-acetate (PMA) + ionomycin stimulation (orange, yellow and green bars respectively). Histograms indicate mean ± sem. (#3 C-T experiments; #2 UI-T experiments; #3 PB experiments), no symbol corresponds to: p=n.s. (Mann-Whitney U-test, for all comparisons).

As depicted in **Figure 8**, progenies exhibited a broad range of cytokine production in basal conditions, which could be further enhanced upon stimulation, aligning with the functional characteristics of Th1, Th1*, and Th17 cells. Notably, the supernatants demonstrated production of IL-2, TNFa, IFN-g, IL-5, IL-13, IL-9, and IL-17a (**Figure 8**).

**Figure 9:**
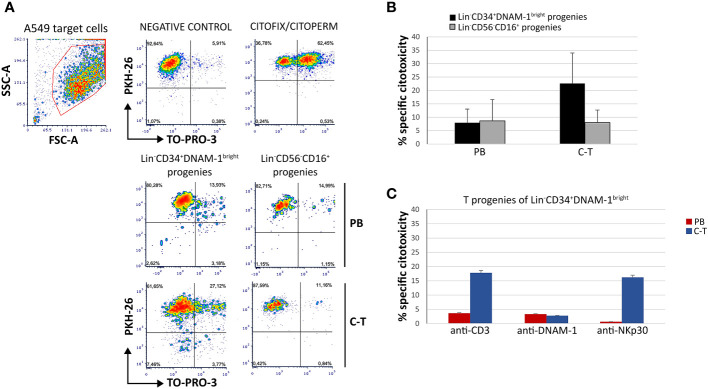
T-cell progenies *in vitro* derived from peripheral blood (PB) and cancer tissue (C-T) CLP cells show cytotoxicity against lung cancer cell line A459 and against P815 target cells in a reverse ADCC assay. **(A)** Flow cytometric cytotoxicity assay of T-cell progenies derived from PB/C-T Lin^-^CD34^+^DNAM-1^bright^ and Lin^-^CD56^-^CD16^+^ cells against A549 cell line targets at 6 hours with effector/target ratio 1:1. FSC-A, forward scatter area; SSC-A, side scatter area. **(B)** Histograms (mean ± sem) indicate the percentage of specific cytotoxic activity against A549 target cells of T-cell progenies derived from PB and C-T Lin^-^CD34^+^DNAM-1^bright^ (black columns) and Lin^-^CD56^-^CD16^+^ (grey columns) cells. Representative of 3 experiments.p=n.s. **(C)** Flow cytometric cytotoxicity assay of T-cell progeny derived from PB (red columns) and C-T (blue columns) Lin^-^CD34^+^DNAM-1^bright^ cells against P815 target cells at 6 hours with effettor/target ratio 1:1. Redirected triggering was obtained by addition of anti-CD3, anti-DNAM-1 and anti-Nkp30 mAbs. Boxes indicate mean ± sem; n= 2 experiments, duplicate wells. no symbol corresponds to: p=n.s., Mann-Whitney U-test.

To additionally evaluate the functionality of T progenies derived *in vitro* from their highly purified precursors, a cytotoxicity assay was performed using a lung cancer target cell line A549 or a mouse FcγR^+^P815 cells in the presence of mAbs specific for triggering receptors, in a redirected killing assay (reverse ADCC). Double staining with PKH-26 and TO-PRO-3 was used to detect killed target cells that appear PKH-26^+^TO-PRO-3^+^ (**Figure 9A**).

T progenies exhibited cytotoxicity towards A549 targets at an effector-to-target ratio of 1:1. Those generated *in vitro* from Lin^-^CD34^+^DNAM-1^bright^ cells purified from cancer tissue displayed significantly greater cytotoxicity compared to those derived from tissue Lin^-^CD56^-^CD16^+^ cells and from peripheral blood (PB) (**Figure 9B**). In the redirected killing assay, T cell progenies from tissue-derived CLPs exhibited potent direct cytotoxic activity upon activation via CD3 and NKp30, outperforming those derived from PBMCs (**Figure 9C**).

Taken together these results indicated that T cell progenies derived from inflammatory precursors purified from blood or from inflamed tissues are functional, produce cytokines consistent with different functional polarization and may be cytotoxic by engagement of their activating receptors.

## Discussion

In the present work we provide evidence that in patients with advanced stage NSCLC, baseline circulation of inflammatory CLP released from BM (1){Bozzano, 2015 #8} is low and increases following the initiation of CT/IT. In addition, these cells enter peripheral tissues including cancer tissue where they are found after chemokine-selective migration and give rise *in vitro* to a majority of functionally active T cells co-expressing activating NK cell receptors.

The finding of a normal baseline circulation of inflammatory precursors in PBMC of patients with advanced stage NSCLC and their increase following CT/IT are in line with the notion that the inflammatory effect of the tumor on the surrounding tissue milieu favors tumor growth and prevents or dampens effective immune responses. Chronic inflammatory conditions may increase the risk of developing cancer (35, 36) and of cancer progression (37). The inflammation orchestrated by the tumor is aberrant and promotes the recruitment and/or the induction of cells that, besides having a role in the direct promotion of the tumor progression, are also endowed with immunosuppressive properties (38). Indeed, patients with NSCLC and little inflammation at baseline before CT/IT have higher progression-free survival (39).

The present observation of a selective increase of CD34^+^DNAM^-^1^bright^CXCR4^+^ after CT/IT, is in line with the inflammatory effects of CT/IT leading to recruitment of inflammatory precursors to the inflamed peripheral tissues and with previous reports where lymphoid precursors including CD34^+^ HSCs increase in the bone marrow, circulate at higher frequency and seed into peripheral tissues (17–20) to replenish immune cells upon increased peripheral turnover (21–25, 40, 41).

Tissue seeding of inflammatory precursors in this case is supported by recovery of precursors in all samples, by chemokine receptor enrichment in tissues favoring CXCR3^+^, CXCR1^+^ and CXCR3^+^ cell entry, by their consistent expression of DNAM-1 and CXCR4 in tissues, and by the similar pattern of progeny generation with characteristics of a T-cell-skewed common lymphocyte precursor both in PBMC and in lung tissues.

The observed increase in CD34^+^DNAM^-^1^bright^CXCR4^+^ cells in PBMC in patients with lung cancer following CT/IT has characteristics of a standard stereotyped defense reaction to inflammatory stimuli (40). This increase is in line with those so far reported during conditions including chronic infection (HIV, HCV) (1, 28), acute infection (SARS-CoV-2) (27), CMV reactivation (10) and persistent/recurrent inflammation (COPD,PAPA syndrome) (1). Notably, CT/IT uniquely triggered an increase in CD34^+^DNAM^-^1^bright^CXCR4^+^ cells, while conventional CD34^+^DNAM-1^-^CXCR4^-^ and also Lin^-^CD56^-^CD16^+^CD7^-^ precursors remained unaltered, This observation supports the hypothesis that CD34^+^DNAM-1^bright^CXCR4^+^ cells may originate from a distinct mechanism that involves selective recruitment from the bone marrow (BM) in response to CT/IT-induced inflammation.

This view is also suggested by the difference in CD34^+^DNAM-1^bright^CXCR4^+^ cell progeny phenotype detected in lung tumor (85-90% T-cells) compared to the considerably lower one (20-30%) reported in patients with infection (CMV, HIV, HCV) (1, 10). These observations could be explained by the hypothesis that different subsets of inflammatory CD34^+^DNAM-1^bright^CXCR4^+^ exist in the BM, that different inflammatory signatures (e.g. tumor vs. infection, acute vs- chronic inflammation) may induce a different upstream precursor development and/or their skewed exit from the BM. Clearly, additional work is needed to further address these points and verify this hypothesis.

With regard to the progenies derived from inflammatory precursors entering lung tissue, also in this case we confirmed previous reports of absent growth of myelomonocytic cells (1, 10) under the same culture conditions that allow growth of NK- and myelomonocitic progenies using cord-blood derived CD34^+^ cells (42, 43). Analysis of maturing T cells *in vitro* after purification of tissue precursors showed that these cells have the ability to produce Th1, Th17 and Th2 cytokines *in vitro*, with a predominant representation of cells with the potential to produce IFNγ (Th1 and Th1*). Interestingly, characterization of these T cell progenies revealed their consistent expression of NK cell activating (NKG2D, DNAM-1, NKp30 that appear to confer HLA-independent function when crosslinked. Although the present analysis was limited in T cell clonality and specificity, according to NK cell receptor expression and function and to Th1 and Th17 cytokine production it appears that these T cell progenies could represent innate-T cells that may be triggered in a TCR-independent fashion. This may be relevant particularly in tumor areas and in patients in whom tumor antigens are elusive or with low HLA-Class I molecule expression.

The extent to which the present *in vitro* differentiation system accurately replicates the diverse conditions of tissues or blood, including the variable levels of local cytokines, chemokines, and stromal factors, remains to be determined. When considering the impact of tissue-specific conditioning on CLP differentiation, two important considerations should be taken into account. Firstly, all cells analyzed were rigorously purified from distinct tissues after a prolonged period of exposure to their local environment. This suggests that the purified precursors had ample opportunity to be conditioned by local cytokines/factors. However, despite this, their extraction and subsequent *in vitro* culture yielded similar progenies in both tissue and peripheral blood mononuclear cells (PBMCs). Furthermore, utilizing the same cytokine cocktail employed for NK cell development from CD34^+^ hematopoietic stem cells *in vitro* resulted in the predominant differentiation of T cells, suggesting that the ability of inflammatory CLPs to follow a specific developmental program may be determined elsewhere (e.g., bone marrow) and that tumor-derived or tissue stimuli may only be able to partially influence their functional trajectory. Future studies are needed to investigate whether and under which experimental conditions different cytokines and stimuli present in tumor tissues might influence CLP development.

Finally, a particularly intriguing question arising from this study concerns the potential relationship between the mobilization and dissemination of inflammatory CLPs to tumor tissues, the extent of tumor lymphocyte infiltration (TIL), and subsequent disease progression. Unfortunately, the current study was not designed to directly assess the association between inflammatory CLPs, TIL, immunoscore, and disease progression in lung cancer patients. Additionally, the limited sample size precluded the detection of associations between inflammatory precursor levels and disease progression. Indeed, information regarding the dissemination of inflammatory CLPs in tumor patients constituted one of the study’s objectives. Consequently, further investigations are warranted to address these questions.

In conclusion, our findings unveil a coordinated inflammatory response to CT/IT that triggers the mobilization and deployment of CXCR4+ precursors to tissues, resulting in the generation of highly functional innate immune cells. This observation provides a new perspective for interpreting the current findings. Tumor microenvironments in the lung favor inflammation by exploiting the SDF/CXCR4 axis, leading to angiogenesis, tumor progression, and metastasis. The SDF/CXCR4 axis plays a crucial role in several mechanisms that promote tumor growth and metastasis (44, 45). Additionally, it contributes to the establishment of immunosuppressive tumor microenvironments, favoring the infiltration of myeloid-derived suppressor cells (MDSCs) and regulatory T cells (Tregs) into tumors (46, 47). Accordingly, the circulation and tissue infiltration of CXCR4^+^ inflammatory precursors following chemotherapy/immunotherapy (CT/IT) in lung cancer patients represent an additional factor that could be exploited to enhance cancer monitoring and immunotherapy strategies by regulating the immune response against tumors.

## Data availability statement

The original contributions presented in the study are included in the article/**Supplementary Material**. Further inquiries can be directed to the corresponding author.

## Ethics statement

The studies involving humans were approved by Comitato Etico Regionale Liguria P.R. 191REG2015. The studies were conducted in accordance with the local legislation and institutional requirements. The participants provided their written informed consent to participate in this study.

## Author contributions

CP: Data curation, Investigation, Writing – original draft, Methodology. FB: Data curation, Investigation, Methodology, Writing – original draft, Conceptualization, Formal analysis. MD: Data curation, Investigation, Methodology, Writing – original draft. GD: Data curation, Investigation, Methodology, Conceptualization, Formal analysis, Writing – original draft. FA: Data curation, Formal analysis, Investigation, Methodology, Writing – original draft. EMu: Data curation, Formal analysis, Investigation, Methodology, Conceptualization, Writing – original draft. EMa: Data curation, Formal analysis, Investigation, Methodology, Writing – original draft. FM: Data curation, Formal analysis, Investigation, Methodology, Conceptualization, Writing – original draft. AH: Data curation, Formal analysis, Investigation, Methodology, Writing – original draft. GP: Data curation, Formal analysis, Investigation, Methodology, Conceptualization, Writing – original draft. MT: Conceptualization, Data curation, Formal analysis, Investigation, Methodology, Supervision, Validation, Writing – original draft. CG: Conceptualization, Data curation, Formal analysis, Investigation, Methodology, Writing – original draft. LM: Conceptualization, Data curation, Formal analysis, Investigation, Methodology, Writing – original draft, Funding acquisition, Resources, Supervision. AD: Conceptualization, Data curation, Formal analysis, Funding acquisition, Investigation, Resources, Supervision, Writing – original draft, Validation, Visualization, Writing – review & editing.

## Supplementary material

Supplementary Figure 1 Flow cytometric analysis of chemokine receptor expression on circulating and tissue-derived inflammatory precursors. **(A)** Flow cytometric analysis of CXCR3, CXCR1, CXCR3, CD62L and CCR7 chemokine receptor expression on Lin^-^CD34^+^DNAM-1^bright^ (Upper row) or Lin^-^CD56^-^CD16^+^CD7^-^ (Lower row) PBMC in cancer patients. Representative of 8 experiments. **(B)** Flow cytometric analysis and gating strategy to identify CXCR3, CXCR1, CXCR3, CD62L and CCR7 chemokine receptor expression on Lin^-^CD34^+^DNAM-1^bright^ CXCR4^+^ (Upper row) or Lin^-^CD56^-^CD16^+^CD7^-^ CXCR4^+^ (Lower row) in cancer-tissue. Among CD3/CD14/CD19/CD20/CD56 negative cells, Lin^-^CD34^+^DNAM-1^bright^ precursors cells appear in the CD16^-^ cell gate and Lin^-^CD56^-^CD16^+^CD7^-^ precursors in the CD16^+^ cell gate. Representative of 15 experiments.

Supplementary Figure 2 Phenotypic characterization by flow cytometry of CD4^+^ T-cell progenies derived from PB, C-T and UI-T CLPs in a representative patient. Cells are gated on CD8^-^CD19^-^CD25^-^CD56^-^CD45RA^+^ cells. Analysis showed T helper cell subsets existence in *in vitro* cultured progenies Declaration of Generative AI and AI-assisted technologies in the writing process: none.
