# Supplementary material for: CD34+DNAM-1brightCXCR4+ haemopoietic precursors circulate after chemotherapy, seed lung tissue and generate functional innate-like T cells and NK cells
Source: Front Immunol. 2024 Feb 8;15:1332781. doi: 10.3389/fimmu.2024.1332781 (PMC10881815; doi:10.3389/fimmu.2024.1332781)
Supplement: Supplementary file 1 [file DataSheet_1.docx]

# SUPPLEMENTARY

**FLUOROCHROME-CONJUGATED mABs**

mAbs anti- human Lineage Cocktail 2 (lin2) (CD3, CD14, CD19, CD20, CD56) FITC: BD Pharmigen, San Jose, CA, USA CLONE NCAM16.2 CAT 643397

mAbs anti- human CD16 PE: BD Pharmigen, San Jose, CA, USA CLONE 3G8 CAT 557758

mAbs anti- human CD16 APCh7 BD CAT 560195 clone 3G8

mAbs anti- human Perforin PE BioLegend, San Diego, CA, USA CLONE dG9 CAT 308106

mAbs anti- human CD7 BV711 BD Pharmigen, San Jose, CA, USA CLONE M-T701 CAT 564018

mAbs anti- human CD226 BV786 BD Pharmigen, San Jose, CA, USA CLONE DX11 CAT 742497

mAbs anti- human CD34 PeCy7 BD Pharmigen, San Jose, CA, USA CLONE 581 CAT 560710

mAbs anti- human CD34 FITC BD Pharmigen, San Jose, CA, USA CLONE 581 CAT 555821

mAbs anti- human CD3 FITC BioLegend, San Diego, CA, USA CLONE HIT3A CAT 300306

mAbs anti- human CXCR4 Purified: BD Pharmigen, San Jose, CA, USA CLONE 12G5 CAT 555972

mAbs anti- human CD4 BV605 BD Pharmigen, San Jose, CA, USA CLONE RPA-T4 CAT 562658

mAbs anti- human CD4 APCH7 BD Pharmigen, San Jose, CA, USA CLONE RPA-T4 CAT 560158

mAbs anti- human CD8 bv650 BD Pharmigen, San Jose, CA, USA CLONE RPA-T8 CAT 563821

mAbs anti- human CD3 BV510 BD Pharmigen, San Jose, CA, USA CLONE UCHT1 CAT 563109

mAbs anti- human CD14 BV 510 BD Pharmigen, San Jose, CA, USA CLONE M5E2 CAT 561391

mAbs anti- human CD19 BV 510 BD Pharmigen, San Jose, CA, USA CLONE SJ25C1 CAT 562947

mAbs anti- human CD56 PeCy7: Immunotech-Coulter, Marseille, France CLONE N901 Item N°A21692

mAbs anti- human CD56 PeCy5: Immunotech-Coulter, Marseille, France CLONE N901 Item N° A07789

mAbs anti- human CX3CR1 PE: MBL International, MA CLONE 2A9-1, CAT D070-5

mAbs anti- human CXCR1 Purified: Santa Cruz Biotechnology, Inc. USA CAT. sc-7303

mAbs anti- human CXCR3 Purified: R&D System, Minneapolis CLONE 49801, CAT MAB160

mAbs anti- human CD62L Purified: BD Pharmigen, San Jose, CA, USA CLONE DREG-56, CAT 555541

mAbs anti- human CD197(CCR7) BV 421 BD Pharmigen, San Jose, CA, USA CLONE 150503 CAT 562555

mAbs anti- human NKP30 APC BD Pharmigen, San Jose, CA, USA CLONE p30-15 CAT 558408

mAbs anti- human NKp46 BV450 BD Pharmigen, San Jose, CA, USA CLONE 9E2/NKp46 CAT 562099

mAbs anti- human NKg2D BV650 BD Pharmigen, San Jose, CA, USA CLONE 1D11 CAT 563408

mAbs anti- human HLADR PerCP-Cy5.5 BD Pharmigen, San Jose, CA, USA CLONE G46-6 CAT 560652

mAbs anti- human NKG2A BV711 BD Pharmigen, San Jose, CA, USA CLONE 131411 CAT 747919

mAbs anti- human TCR Vα24 APCVIO770 Miltenyi Biotec, Bergisch Gladbach, Germany CLONE REA948 CAT 130-115-735 mAbs anti- human CD45RA APCH7 BD Pharmigen, San Jose, CA, USA CLONE HI100 CAT 560674

mAbs anti- human CCR4 PE BD Pharmigen, San Jose, CA, USA CLONE 1G1 CAT 551120

mAbs anti- human CCR4 PE-Cy7 BD Pharmigen, San Jose, CA, USA CLONE 1G1 CAT 561034C

mAbs anti- human CCR10 PERCPC5.5 BD Pharmigen, San Jose, CA, USA, CLONE 1B5 CAT 564772

mAbs anti- human CCR10 PERCPC5.5 BD Pharmigen, San Jose, CA, USA, CLONE 1B5 CAT 563656

mAbs anti- human CD196 (CCR6) BV421, BD Pharmigen, San Jose, CA, USA CLONE 11A9 CAT 562515

mAbs anti- human CD57APC BD Pharmigen, San Jose, CA, USA CLONE CAT560845

mAbs anti- human NKG2CPurified R&amp;DSystems, Inc. Minneapolis, MN 55413 CAT MAB1381

anti-KIR2DL2/L3/S2 (CD158b1/b2,j), anti-KIR3DL1/S1 (CD158e1/e2), anti-KIR2DL1/S1

(CD158a/h), anti-NKG2A (Z270, IgG1; Z199, IgG2a), and anti-CD85j (F278, IgG1 kindly provided by

Dr. D. Pende), all of which were produced in the laboratory (A. Moretta, Genova, Italy).

# ANTI-ISOTYPE-SPECIFIC GOAT ANTI-MOUSE SECONDARY REAGENTS

Goat Anti-Mouse IgG1 PE conjugated. Beckman Coulter CA, CAT 731840

Goat Anti-Mouse IgG2b FITC conjugated. Southern Biotech, Birmingham, AL, USA **CAT 1090-02**
