# Supplementary figures and images for: CD34+DNAM-1brightCXCR4+ haemopoietic precursors circulate after chemotherapy, seed lung tissue and generate functional innate-like T cells and NK cells
Source: Front Immunol. 2024 Feb 8;15:1332781. doi: 10.3389/fimmu.2024.1332781 (PMC10881815; doi:10.3389/fimmu.2024.1332781)

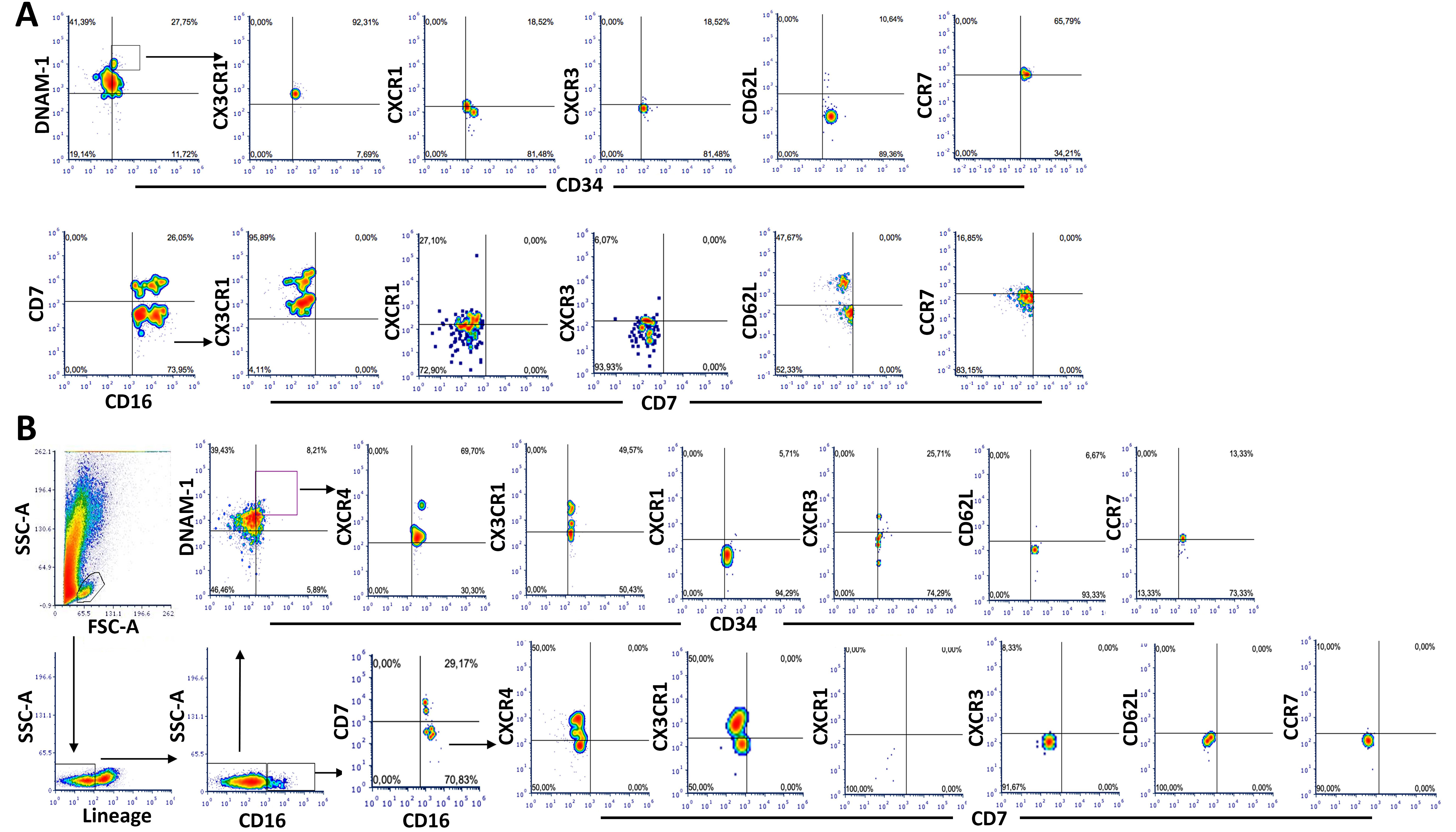

Supplement: Supplementary Figure 1 — Flow cytometric analysis of chemokine receptor expression on circulating and tissue-derived inflammatory precursors. (A) Flow cytometric analysis of CXCR3, CXCR1, CXCR3, CD62L and CCR7 chemokine receptor expression on Lin-CD34+DNAM-1bright (Upper row) or Lin-CD56-CD16+CD7- (Lower row) PBMC in cancer patients. Representative of 8 experiments. (B) Flow cytometric analysis and gating strategy to identify CXCR3, CXCR1, CXCR3, CD62L and CCR7 chemokine receptor expression on Lin-CD34+DNAM-1bright CXCR4+ (Upper row) or Lin-CD56-CD16+CD7- CXCR4+ (Lower row) in cancer-tissue. Among CD3/CD14/CD19/CD20/CD56 negative cells, Lin-CD34+DNAM-1bright precursors cells appear in the CD16- cell gate and Lin-CD56-CD16+CD7- precursors in the CD16+ cell gate. Representative of 15 experiments. [file Image_1.jpeg]

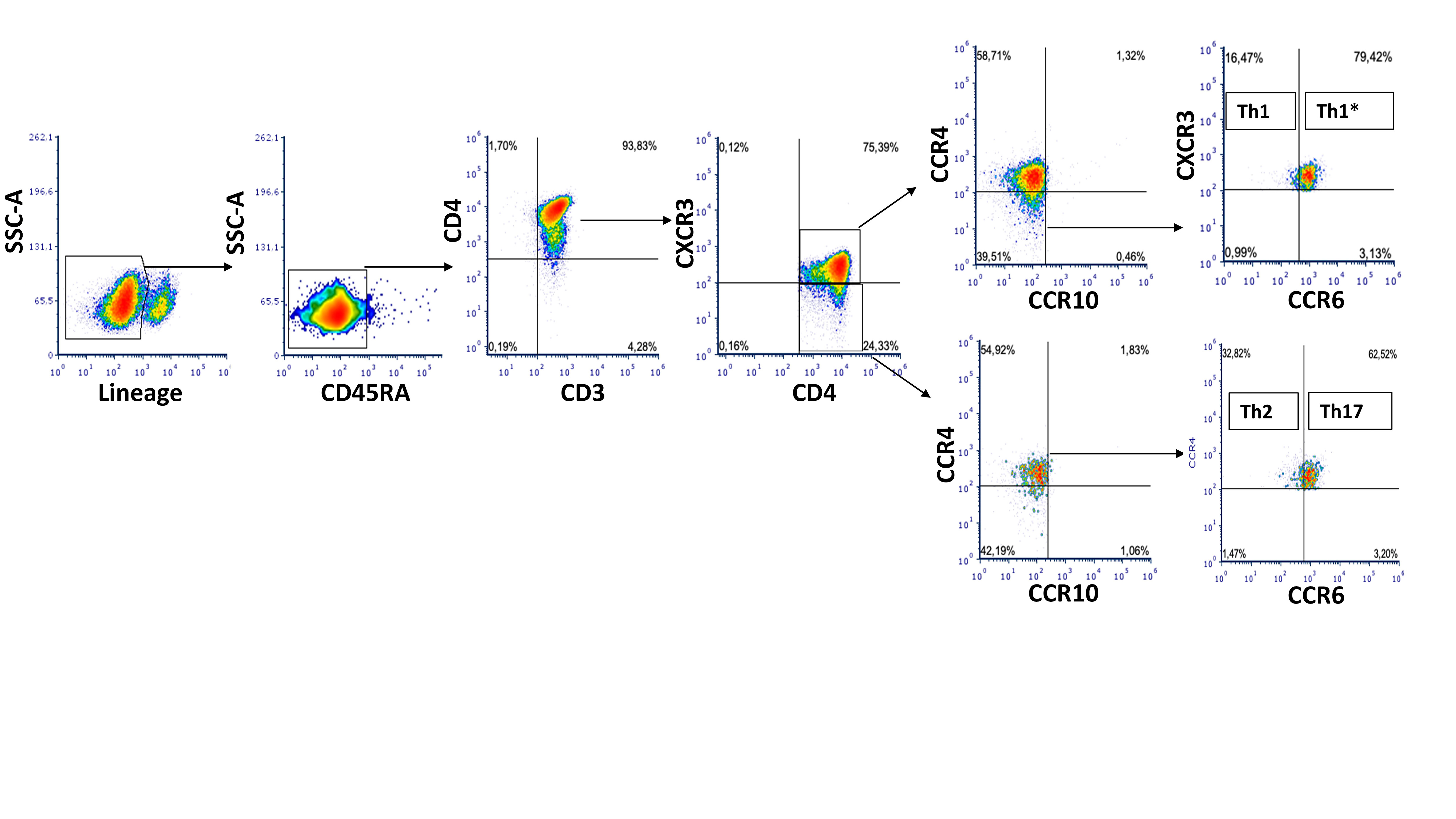

Supplement: Supplementary Figure 2 — Phenotypic characterization by flow cytometry of CD4+ T-cell progenies derived from PB, C-T and UI-T CLPs in a representative patient. Cells are gated on CD8-CD19-CD25-CD56-CD45RA+ cells. Analysis showed T helper cell subsets existence in in vitro cultured progenies Declaration of Generative AI and AI-assisted technologies in the writing process: none. [file Image_2.jpeg]
